# Artificial Intelligence in Medicine: Text Mining of Health Care Workers’ Opinions

**DOI:** 10.2196/41138

**Published:** 2023-01-27

**Authors:** Pascal Nitiéma

**Affiliations:** 1 Department of Information Systems Arizona State University Tempe, AZ United States

**Keywords:** artificial intelligence in medicine, artificial intelligence in health care, artificial intelligence, AI, algorithm, machine learning, deep learning, structural topic modeling

## Abstract

**Background:**

Artificial intelligence (AI) is being increasingly adopted in the health care industry for administrative tasks, patient care operations, and medical research.

**Objective:**

We aimed to examine health care workers’ opinions about the adoption and implementation of AI-powered technology in the health care industry.

**Methods:**

Data were comments about AI posted on a web-based forum by 905 health care professionals from at least 77 countries, from May 2013 to October 2021. Structural topic modeling was used to identify the topics of discussion, and hierarchical clustering was performed to determine how these topics cluster into different groups.

**Results:**

Overall, 12 topics were identified from the collected comments. These comments clustered into 2 groups: impact of AI on health care system and practice and AI as a tool for disease screening, diagnosis, and treatment. Topics associated with negative sentiments included concerns about AI replacing human workers, impact of AI on traditional medical diagnostic procedures (ie, patient history and physical examination), accuracy of the algorithm, and entry of IT companies into the health care industry. Concerns about the legal liability for using AI in treating patients were also discussed. Positive topics about AI included the opportunity offered by the technology for improving the accuracy of image-based diagnosis and for enhancing personalized medicine.

**Conclusions:**

The adoption and implementation of AI applications in the health care industry are eliciting both enthusiasm and concerns about patient care quality and the future of health care professions. The successful implementation of AI-powered technologies requires the involvement of all stakeholders, including patients, health care organization workers, health insurance companies, and government regulatory agencies.

## Introduction

### Background

Artificial intelligence (AI) can be described as a set of technologies that can perform tasks usually associated with human cognitive functions such as learning, pattern recognition, reasoning, and problem solving. Many tasks performed by workers in their job involve the use of these cognitive functions, and as such, these tasks can be performed entirely or partially by AI. AI in health mainly involves developing algorithms and predictive models that can be integrated into the delivery of routine care to patients, health care organization operations, health promotion, or public health programs. Current computing capabilities allow harnessing and processing high volumes of data points to build algorithms that can assist health care workers in patient care decision-making. Applications of AI in health include self-referral, in which individuals enter personal health information into AI applications to obtain recommendations on whether they should seek care for their condition; triage of patients in health facilities; image-based diagnosis in radiology and pathology; treatment recommendations to clinicians through clinical decision support systems; fraud detection by identifying unusual patterns in claim data; and disease surveillance and outbreak detection by scanning population health data. Many studies have reported how algorithms perform better than health care professionals in diagnosing, monitoring, or finding adequate treatment for certain health conditions, including assessment of skin lesions [1], lung cancer screening [2], and cancer survival prognosis [3]. Despite this encouraging news about the performance of AI in health care, this technology is set to disrupt the routine operations in patient care. The adoption of new technologies can disrupt organizations’ routines, especially those performed by interdependent organizational members [4]. These disruptions can lead to reluctance among health care professionals to use AI-powered tools in their job.

A technology can only yield expected results if used by members of the organization. Hence, it is useful to investigate the comments about the introduction of AI in the health care industry. A useful model for examining how individuals adapt to new technologies in their working environment is the coping model of user adaptation (CMUA) [5], which was derived from the transactional model of stress and coping [6]. CMUA describes 2 types of responses depending on whether the users perceive the new technology as a threat that prevents them from attaining their goal or as an opportunity for obtaining the desired outcomes. If an individual perceives a technology as a threat, a distress response ensues, and the individual may react by avoiding or distancing themselves from the technology. However, if the new technology is appraised as an opportunity by the individual, they may take full advantage of it to reap the expected benefits [5].

### Objective

Although there have been some studies conducted to examine health care professionals’ attitudes and beliefs about the impact of AI technologies on their profession and the delivery of care to patients, most of these studies have been survey-based with predesigned questionnaires. Such studies do not provide the opportunity to compile a comprehensive list of workers’ expectations and concerns. The advantage offered by the analysis of unstructured textual data collected from web-based forums over structured survey research is that open forums contain opinions and comments spontaneously expressed by participants, without the constraints of survey items or the influence of the investigator’s research framework. Such extemporaneous comments may help uncover new information and previously unknown features about the topic of interest. The purpose of this study was to examine health care professionals’ opinions about the adoption of AI technologies by their organizations.

## Methods

### Data Collection

We collected data from Medscape (WebMD), a web-based platform dedicated to health care professionals. According to the digital intelligence company Similarweb [7], Medscape had 4.2 million monthly visits from 2.3 million monthly unique visitors during the spring of 2021. During that period, 48% of the visits were from the United States, 4.5% from the Philippines, 3.8% from the United Kingdom, 3.5% from India, 3% from Canada, 2.9% from Australia, and 1.9% from Malaysia. We used the keywords, *artificial intelligence, AI, machine learning, deep learning,* and *neural network*, to identify the comments of interest. The collected data spanned from May 2013 to October 2021. The collected comments were limited to the English language. In addition to comments, the collected data included the commenters’ occupation, medical specialty, and country or state of residence, as reported by these individuals on their profiles.

### Ethical Considerations

As the data collected for this study were publicly available, the institutional review board of the University of Oklahoma determined that ethics approval was not necessary for the study.

### Data Analysis

#### Sentiment Analysis

A sentiment score is a value that represents the valence and magnitude of emotions in a word, a sentence, or a group of sentences. A negative score denotes a negative emotion (eg, sadness, anger, frustration, and anxiety), and a positive score denotes a positive emotion (eg, joy, excitement, pleasure, and pride). The sentiment scores of the comments were computed with the R package, *sentimentr* (version 2.9.0), which accounts for valence shifters (eg, good vs not good), amplifiers (eg, good vs very good), and deamplifiers (eg, good vs barely good) [8].

#### Structural Topic Modeling

**Figure 1:**
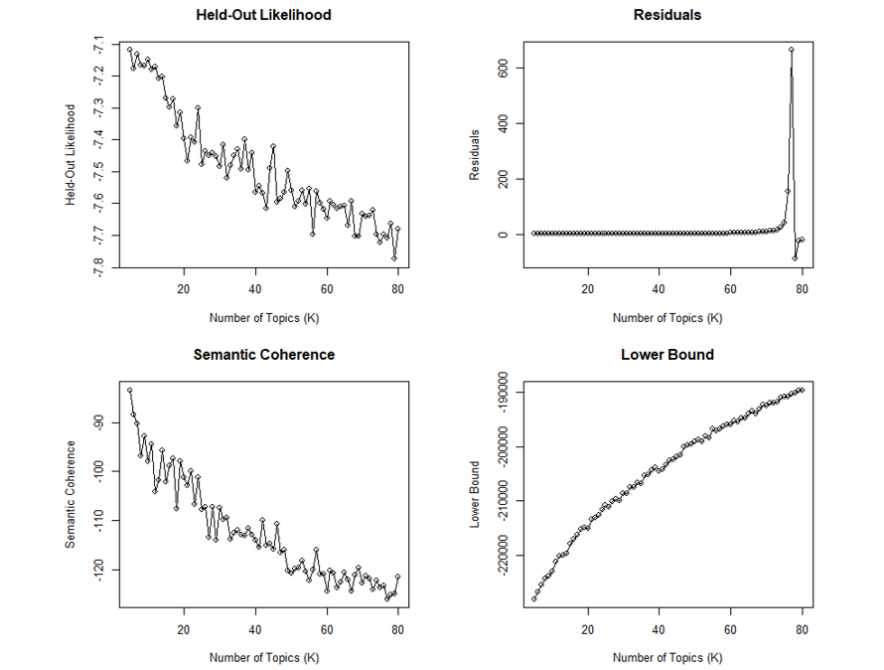
Diagnostic values according to number of topics.

After performing sentiment analysis, the textual contents of the comments were preprocessed using the *tm* package [9]. Stop words were removed using the System for the Mechanical Analysis and Retrieval of Text stop word list. The words were stemmed to obtain a more compact corpus. Structural topic modeling was then performed to identify the topics that were discussed across the comments collected. Structural topic modeling allows the integration of information about the documents (metadata) into the topic modeling process [10]. Several studies have used topic modeling to examine workers’ opinions on specific topics [11-13]. The following metadata were used as covariates for estimating topic prevalence: year of comment, commenter’s residence (United States vs non–United States), commenter’s occupation (physician, nurse, health administrator, or other), and the sentiment score of the comment. On the basis of the values for held-out likelihood, semantic coherence, residuals, and lower bound, the appropriate number of topics was determined to be between 10 and 20 (Figure 1). The appropriate number of topics to be extracted was determined by selecting the model that yielded both highest semantic coherence and exclusivity values. Thus, the number of 12 topics was selected for modeling. Spectral initialization was used for topic modeling. The model was run through 5000 iterations and reached convergence. The final model comprised 12 topics, 1126 documents (n=10, 0.89% of the documents were excluded for not containing any word after corpus preprocessing), and a 2897-word dictionary. The 12 topics were labeled by examining the word stems with the highest frequency; the highest value for the frequency and exclusivity (FREX) metric, a measure that accounts for word exclusivity and frequency [14]; and the highest score values (a metric that gives more weight to words that are less frequent in other topics) [15]; and finally, by examining the quotes from each topic. The maximum a posteriori estimates of the identified topics were used to compute the pairwise correlations among the identified topics. A threshold of *r*=0.01 was used to determine whether 2 topics were correlated. Hierarchical clustering (threshold=1.50) was performed to identify clusters of topics. Furthermore, multidimensional scaling of the identified topics was conducted to visualize the distances among these topics. Topic modeling and multidimensional scaling were conducted using the R package, *stm* [15], whereas hierarchical clustering of topics was performed using the R package, *stmCorrViz* [16].

## Results

### Overview

**Figure 2:**
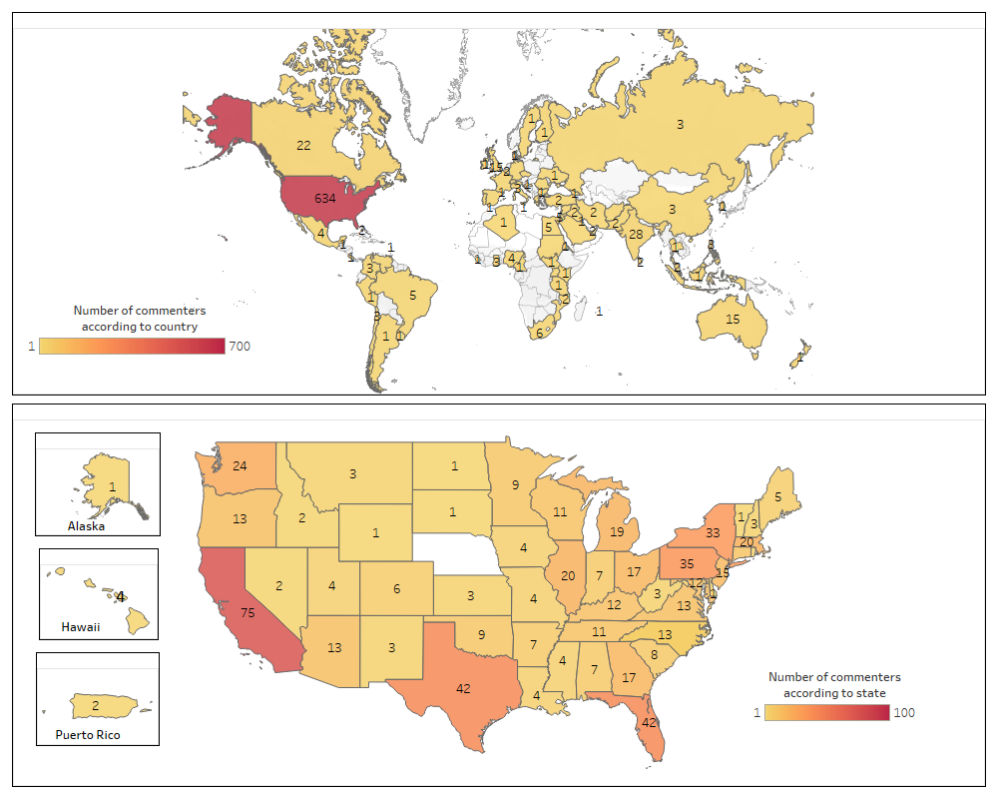
Geographic distribution of commenters.

A total of 1136 comments made by 905 health care professionals were collected. The length of the comments ranged from 1 to 616 (mean 76.8, SD 80.8; median 50) words. The 905 health care professionals were from at least 77 countries on 6 continents (Africa, Asia, Oceania, Europe, North America, and South America). Of the 905 individuals, most (n=634, 70.1%) resided in the United States, 28 (3.1%) in India, and 22 (2.4%) in Canada. Figure 2 presents the distribution according to country of residence of health professionals who posted the comments on AI, and Table 1 shows the distribution of these health professionals according to region of residence and occupation.

**Figure 3:**
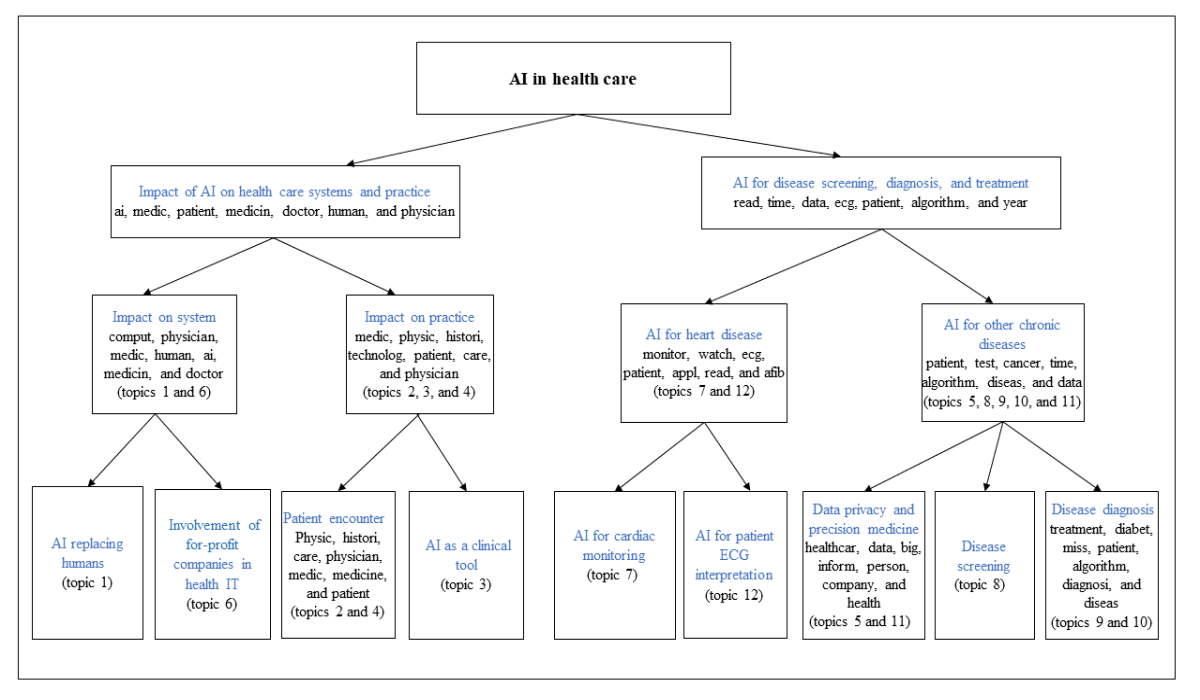
Hierarchical clustering of identified topics (threshold=1.50). AI: artificial intelligence; ECG: electrocardiogram.

The identified topics are presented in Table 2, and the quotes for each topic are provided in Multimedia Appendix 1. There was no time trend in the prevalence of each of the extracted topics (all *P* values were >.05, with 2013 as the reference year). Only 2 estimates of the topics’ pairwise correlations were >0.01. Topic 6 (involvement of for-profit companies in health IT) was correlated with topic 1 (AI replacing humans; *r*=0.04), and topic 4 (patient care by health care professionals) with topic 2 (AI and traditional diagnostic procedures; *r*=0.02). The 12 topics clustered into two groups: (1) impact of AI on health care system and practice and (2) AI as a tool for disease screening, diagnosis, and treatment (refer to Figure 3). The 2 groups of topics are described in the following sections.

**Table 1:** Characteristics of health care workers who posted the comments (N=905).

Characteristics			Individuals, n (%)
**Region**			
	North America^a^	656 (72.5)	
	Asia	70 (7.7)	
	Europe	58 (6.4)	
	Africa	32 (3.5)	
	Central or South America	29 (3.2)	
	Oceania (Australia or New Zealand)	16 (1.8)	
	Unknown	44 (4.9)	
**Occupation or position**			
	Physician	591 (65.3)	
	Nurse	93 (10.3)	
	Health administration	41 (4.5)	
	Other health occupation	140 (15.5)	
	Unknown	40 (4.4)	

^a^The United States: 634/905, 70.1%; Canada: 22/905, 2.4%.

**Table 2:** Description of identified topics.

Topic and label	Percentage of corpus (%)	Sentiment score coefficient estimate	*P* value	Top words and word stems		
				Highest probability^a^	FREX^b^	Score^c^
1—AI^d^ replacing humans	11.6	−0.05	.02	ai, human, doctor, physician, comput, medicin, and replac	human, ai, empathi, robot, job, write, and compass	human, ai, doctor, robot, empathi, radiologist, and radiolog
2—AI and classical medical examination	10.3	−0.01	.70	histori, physic, patient, physician, exam, technolog, and diagnosi	histori, physic, exam, examin, affect, student, and differenti	exam, histori, physic, affect, examin, listen, and student
3—AI as a clinical tool	10.1	0.20	<.001	clinic, tool, clinician, learn, watson, medic, and machin	watson, clinician, tool, imag, clinic, integr, and nice	watson, skill, integr, clinic, imag, brain, and augment
4—Patient care by health care professionals	9.5	0.16	<.001	patient, care, doctor, provid, medic, nurs, and time	nurs, quick, practition, heal, hold, retir, and back	nurs, care, pa, doctor, quick, store, and lab
5—Data privacy	8.5	0.01	.47	data, big, inform, compani, individu, insur, and point	compani, collect, correl, big, data, sell, and mental	data, big, collect, correl, compani, privaci, and network
6—Involvement of for-profit companies in health IT	8	−0.01	.72	medic, physician, good, patient, medicin, system, and googl	govern, googl, well, judgement, burnout, profit, and pull	googl, govern, judgement, profess, burnout, psychiatrist, and profit
7—AI for cardiac monitoring	7.7	0.01	.62	watch, monitor, appl, devic, peopl, patient, and afib	appl, kardia, watch, monitor, wearabl, implant, and older	watch, appl, monitor, afib, kardia, rhythm, and devic
8—Disease screening	7.1	−0.09	<.001	cancer, test, predict, ai, posit, treatment, and year	alzheim, breast, cancer, microsoft, death, fals, and tumor	cancer, alzheim, cognit, death, breast, fals, and microsoft
9—AI for diabetic retinopathy	7	−0.05	.01	algorithm, patient, diabet, care, provid, eye, and miss	ophthalmologist, retin, realli, algorithm, eye, diabet, and child	algorithm, diabet, eye, ophthalmologist, retinopathi, ear, and retin
10—AI and medical diagnostic procedure	6.8	−0.21	<.001	patient, diagnosi, diseas, problem, diagnos, treatment, and time	sensor, investig, wrong, also, breath, sound, and tell	breath, sensor, investig, spine, fundus, color, and feed
11—Personalized medicine	6.8	0.07	.01	healthcar, health, technolog, cost, person, medicin, and care	idea, forev, lifestyl, stress, gp, ethnic, and level	health, diabet, forev, gp, stress, hbac, and cost
12—AI for patient ECG^e^ interpretation	6.7	−0.04	.07	ecg, read, comput, year, cardiologist, afib, and interpret	cardiologist, alivecor, ecg, read, cardiolog, week, and interpret	ecg, cardiologist, afib, alivecor, cardiolog, read, and atrial

^a^List of the most frequent word stems in the topic.

^b^FREX: frequency and exclusivity; list of word stems with the highest FREX values.

^c^List of word stems with the highest score.

^d^AI: artificial intelligence.

^e^ECG: electrocardiogram.

### Impact of AI on Health Care System and Care Practice

#### Overview

The topics on the impact of AI on health care systems and care practices included comments describing how AI may alter the current health care system and practices of patient care. Overall, these topics represented 49.5% of the corpus. The comments from these topics clustered into two subgroups: (1) the impact of AI on health care systems and (2) the impact of AI on care practice.

#### Impact of AI on Health Care Systems

The topics on the impact of AI on health care systems included the topic of AI replacing humans (topic 1; 11.6% of the corpus), which was associated with a negative sentiment score. Some health care workers were concerned that the adoption of AI in health care will lead to the elimination of jobs in the industry, as illustrated in the following comments:

AI will replace both the office workers and the physician. It’s coming.

August 2018

We are all entirely replaceable and whether it happens in 20 years or in 200 years it will definitely happen.

November 2018

AI will replace physicians and all other clinicians in less than 50 years. No question.

November 2018

I think AI will be a game changer for future physicians and some medical specialties will be completely replaced and others at least will be augmented by AI technology.

November 2018

Once AI becomes cheaper than a clinician, AI and technicians will replace many clinical functions in psychiatry/mental health.

February 2020

Furthermore, some commenters argued that by placing the focus on data and algorithms rather than the patient-clinician relationship, AI will alter and devalue the “art of medicine.” A physician commented the following:

I wonder if a machine can teach the Art of Medicine or can it feel compassion, empathy, and cry along with those in grief.

November 2018

However, there was opposition from other commenters against the idea that AI is set to replace humans in the health care industry:

I don’t see any doctors being replaced, but job descriptions may change a little. AI is useful as a tool and a decision aid, but has currently too hard a time of getting the big picture right.

September 2018

I think AI can’t replace doctors since humans can only be understood emotionally by other humans. Also, patients may not trust AI because people believe that machines are more exposed to errors than humans.

November 2018

AI can’t replace the human touch in human medicine. I think there will be a place for them but I really hope that they don’t “replace” doctors in seeing, diagnosing patients.

November 2018

Discussions about the impact of AI on health care also covered the involvement of for-profit companies in medical AI (topic 6; 8% of the corpus). The potential involvement of private companies such as Google, Apple, or Amazon in designing and marketing health care AI elicited concerns from the health care workers. These apprehensions included financial interests trumping principles of equity in access to quality care and total dependence of health care professionals on copyrighted algorithms for delivering patient care. These concerns led a physician to call for the involvement of governments and universities in the development of AI applications for health care:

If the government and university leaders do not get involved today in creating a health AI system that would benefit all, this will become an exclusive domain of Google and Amazon [already working on it] which will mean more profit for them, and less health care equality. In my mind AI is the solution to health care cost and access disparity but the problem will get worse if tech giants will have sole control of it.

July 2019

#### Impact of AI on Care Practice

Topics on the impact of AI on patient care practices included discussions about AI and classical medical examination (topic 2; 10.3% of the corpus), AI as a clinical tool (topic 3; 10.1% of the corpus), and patient care by health care professionals (topic 4; 9.5% of the corpus). Commenters discussing the impact of AI on classical medical diagnostic procedures (topic 2) opined that the adoption of AI will lead clinicians to rely less on history and physical examination of patients as diagnostic methods, which may lead to decrease in the quality of medical care. This viewpoint prompted many commenters to advocate for these classical diagnostic procedures, as illustrated in the following comments:

New technologies may add something to this diagnostic process but at the end of the day we should rely on our composite evaluation of the patient by history and examination in the light of past experience.

March 2016

AI may help with atypical presentations but attention to detail and the basics of history & physical [examination] are irreplaceable.

August 2018

Concerns about the minimization of the role of physical examination in patient care led some commenters to raise the question of legal liability if the lack of physical examination leads to diagnostic errors:

And to suggest that a current physical assessment would no longer be warranted is absurd. I would imagine that this would or could lead to quite a few more lawsuits one day by allowing important and highly pertinent information falling through the cracks.

September 2019

Liability for lawsuits that may be filed by patients who have been treated with recommendations made by AI was also raised:

How do you sue a computer who makes a medical mistake?

February 2018

Who will the patients sue when they are unhappy with outcomes?

November 2018

If your appendicitis did not fall within the detection ability of the algorithms, sorry for you. At first it will be like the auto driving Tesla. There will be a few lawsuits not against the actual computer but against the manufacturer of the computer.

December 2018

### AI as a Tool for Disease Screening, Diagnosis, and Treatment

#### Overview

The second group of identified topics was regarding the use of AI for disease screening, diagnosis, and treatment and included 7 topics, representing 50.5% of the corpus. The pathologies mentioned in these topics were reflective of those for which AI tools were being developed. These pathologies included cancer, diabetes, dementia and cognitive impairment, and various skin and cardiac diseases. The 7 topics in this group clustered into 2 subgroups that are described in the following sections.

#### AI for Chronic Diseases

Comments on the use of AI for screening diagnosis and treatment of chronic diseases included 5 topics: concerns about data privacy (topic 5; 8.5% of the corpus), disease screening with AI tools (topic 8; 7.1% of the corpus), AI for diabetic retinopathy (topic 9; 7% of the corpus), AI and medical diagnostic procedure (topic 10; 6.8% of the corpus), and personalized medicine (topic 11; 6.8% of the corpus).

In this group of topics, the advantages of AI in health care were clearly enunciated, including improved accuracy of diagnostic procedures that rely on digital image analysis (eg, interpretation of medical imaging in radiology, biopsies in pathology, skin lesions in dermatology, and fundus imaging in ophthalmology) and the promotion of precision medicine, that is, the selection of adequate procedures and treatments for specific patients based on their individual characteristics. A physician wrote the following:

Combining a full range of omics data with patient reported data will herald true personalized and precision medicine.

September 2019

However, concerns about the privacy of the data used for developing and using the algorithms were raised:

I am happy to hear about this disruptive and likely helpful effort. I do have concerns about whether the use of this data in this way opens the door to creation and storage of protected health information [PHI]. Simply saying people who value privacy “wouldn’t be on social media” is not adequate. If the type and amount of data is sufficient to meet the standard, proper observation of HIPAA compliance is required according to the Privacy Rule...

January 2019

Our privacy is being eroded day by day. Companies like Google do not have our best interests at heart. That should be of concern to everyone. I’m not willing to trade personal privacy for anything.

February 2019

The same Google with a business model that monetizes, sells and re-sells personal data on a massive scale with no respect, protection or expectation of privacy? Not to mention unintended loss of data through hacking by the millions. How do I opt-out my data? That’s the trouble, I can’t.

February 2019

#### AI for Heart Disease

Discussions about the use of AI tools for heart diseases included 2 topics. The first one, AI for cardiac monitoring (topic 7; 7.7% of the corpus), included comments on the use of AI-powered tools for monitoring individuals to detect arrhythmias such as atrial fibrillation. Many commenters pointed out the practicality of these tools for patient clinical surveillance, especially for individuals with risk factors or a history of heart disease.

The second topic, AI for patient electrocardiogram (ECG) interpretation (topic 12; 6.7% of the corpus), was focused on the use of AI for interpreting ECG. Although the average sentiment score was not significantly different from 0 (score=−0.04; *P*=.07), a frequent thread in the topic was a list of concerns about the accuracy of ECG interpretations by AI-powered tools:

I don’t know what goes into the algorithms for the computer output of today’s ECG machines, but they need a lot of reviewing, probably with a larger database and heuristics...

April 2017

Never I trust ECG reading by machine. Accurate position of electrodes and real analysis of ECG, according to clinical situation and examination are the main keys for ECG interpretation and Diagnosis.

April 2017

However, others commented that AI-powered machines can provide accurate ECG interpretation, and the issue with inaccurate ECG reading may stem from lack of skill set for using the machine appropriately:

If IBM programmed Watson to read ECG and then competed against distinguished ECG cardiologists, I would bet on Watson. Chess is much harder than reading ECG. The fake afib [atrial fibrillation] and its clinical consequences are due to the non-cardiologist MD not having the skillset to over-read the ECG computer.

April 2017

ECG as such today is getting second importance in developed countries and therefore most of the time they are done by less experienced personal.

April 2017

### Distribution of Topics via Multidimensional Scaling

**Figure 4:**
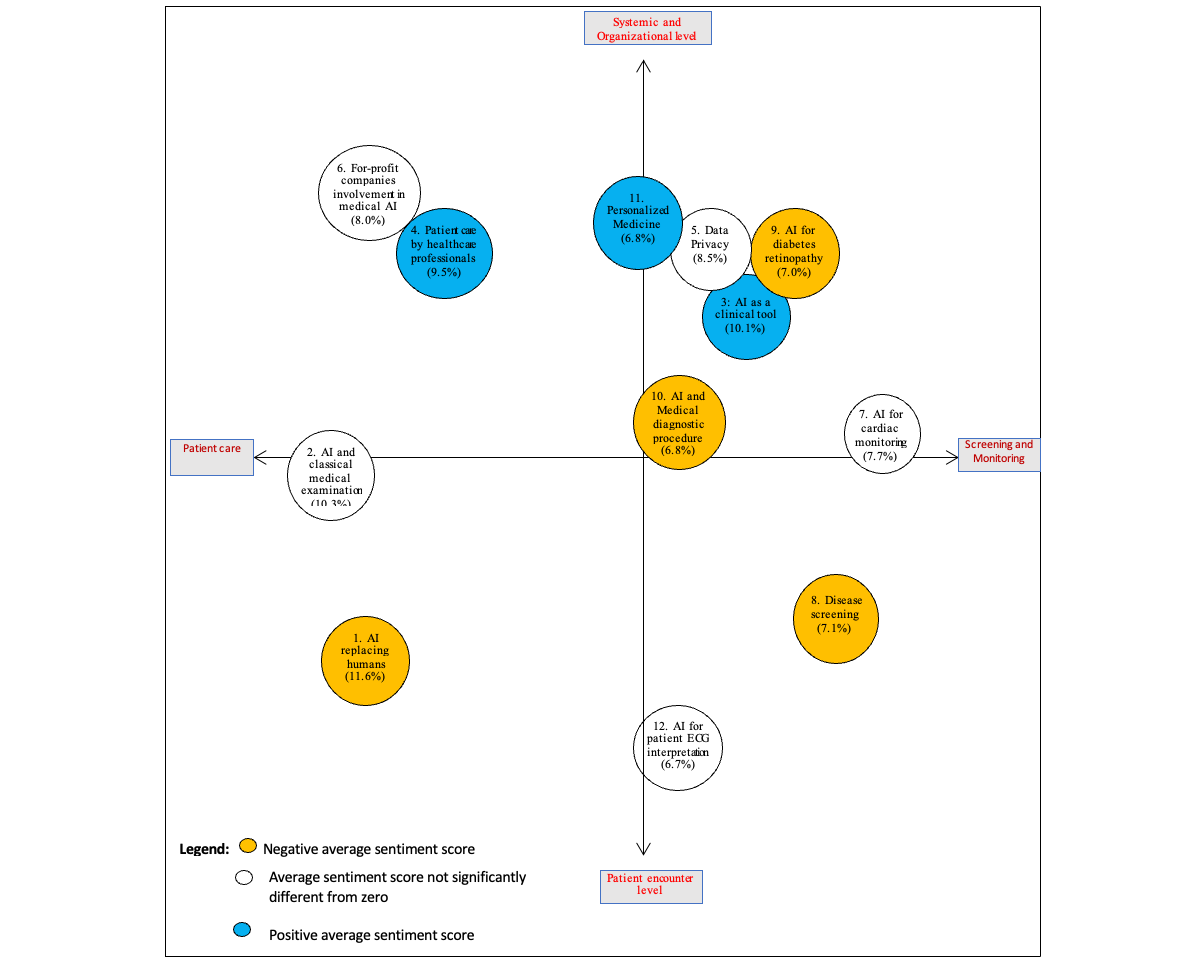
Intertopic distance map obtained via multidimensional scaling. Topic proportions are given within parentheses. AI: artificial intelligence; ECG: electrocardiogram.

Multidimensional scaling identified the 2 axes defining the Cartesian plane over which the 12 topics were distributed and allowed the visualization of the distance between these topics (refer to Figure 4). The x-axis of the plane was described as *patient care—screening and monitoring*, and the y-axis was described as *patient encounter level—systemic and organizational level*. There were more topics in the quadrant, *systemic and organizational level—screening and monitoring* (3 topics fully, 25.6% of the corpus and 3 other topics partially, 21.3% of the corpus), than in any of the 3 other quadrants. All the 3 topics closest to the *patient encounter level* extremity of the y-axis had a negative average sentiment score, whereas all the 3 topics with a positive average sentiment score were close to the *organizational level* extremity.

## Discussion

### Principal Findings

The purpose of this study was to examine health professionals’ opinions about the adoption and implementation of AI applications by health care organizations. The findings can be interpreted through the lens of CMUA, which suggests that users respond differently to a new technology depending on whether they appraise the technology as a threat or as an opportunity [5]. This pattern of threat or opportunity can be found among the topics identified from the collected data. The topics can be grouped into 2 streams: one that described AI as a threat (eg, topic 1: AI replacing humans, topic 8: AI for disease screening, and topic 10: AI and medical diagnostic procedure), reflected in the negative average sentiment scores, and the other that perceived AI as an opportunity for enhancing the quality of health care (topic 3: AI as a clinical tool and topic 11: personalized medicine), as demonstrated by the positive average sentiment scores. The multidimensional scaling of the topics revealed that at least 2 topics with negative average sentiment scores were close to the adoption of AI-powered applications at the patient encounter level. In contrast, topics with positive average sentiment scores were close to the adoption of AI technologies at the organizational level. This finding suggests that the reluctance to adopt AI may be high when AI-powered technology is applied to clinical practice compared with when it is integrated into organizational or system-level operations. In the following sections, we discuss the findings through the 2 identified clusters of topics.

### Impact of AI on Health Care System and Care Practice

The analysis found that the topic of computers replacing humans (topic 1; 11.6% of the corpus) was the most frequently discussed topic regarding AI in health care. This topic was associated with a negative sentiment, hinting that the health care workers who discussed the topic were worried about the negative consequences of workers being replaced by AI applications. Similar concern about job security owing to the adoption of AI has been reported in a sample of clinical laboratory employees [17] and radiographers [18]. The concern about being replaced by AI is not specific to health care workers, and previous literature has reported similar findings among workers in the hospitality industry [19] and among employees performing manual or physical tasks [20]. This concern about job security is a dimension of technostress and has been labeled as *techno-insecurity* [21]. Technostress has been found to reduce job satisfaction and organizational commitment [21]. Similar findings were reported by Brougham and Haar [22], who observed that great awareness about AI, robotics, and algorithms was associated with low organizational commitment, low career satisfaction, and high turnover in a sample of workers in New Zealand. Surprisingly, the awareness about AI, robotics, and algorithms was not associated with job insecurity, thus prompting the authors to speculate that AI may have been interfering with these workers’ career planning (ie, contemplating a change of career) because with these new technologies, the workers’ type of job or industry may entirely disappear [22]. Hence, the adoption of AI by health care organizations to improve patient care quality and organizational outcomes can add to the stress already experienced by health care workers.

Another topic amply discussed by commenters is the impact of AI on traditional medical examination such as patient medical history enquiry and physical examination (topic 2; 10.3% of the corpus). Physical examination is a core component of classical medical diagnostic procedure and is always emphasized during the training of clinicians. Therefore, it is not surprising that performing a physical examination has become part of the identity of clinicians [23]. Hence, any technology that is perceived to reduce the importance of physical examination can also be perceived as altering the clinician’s identity. This concern about technology minimizing the importance of physical examination is not specific to AI but seems to be associated with the adoption of new technologies in general, including imaging diagnostic procedures and biomolecular testing [24]. Physical examination is also perceived by health care workers to be an important element for establishing patient-clinician relationship [23]. Some commenters opined that, by de-emphasizing the importance of physical examination, AI may negatively affect the quality of this relationship, with some suggesting that AI may reduce the importance of human touch and empathy in patient care. The concern about the erosion of the patient-clinician relationship is not specific to AI and has been mentioned with the adoption of other ITs in health care (eg, electronic health records), as reported by previous studies [25-28].

Some commenters raised concerns about the legal liability involving the use of AI in health care. Gray areas exist, and tort law regarding medical malpractice needs to be updated to address lawsuits that may arise from the use of AI. Apportioning liability may be challenging, especially for algorithms that have been developed through neural networks and constitute a black box for both the manufacturer and the clinician using them. In neural networks, algorithms for classification or decision-making are built by feeding the software with data that the analyst considers relevant to the context. Using the provided data, the software yields an algorithm that the analyst, manufacturer, and clinician cannot fully understand [29,30]. Only the prediction or classification accuracy of the algorithm can be evaluated. In addition, such algorithms are *autonomous* and change over time as they are fed with more data on the field. That is, the algorithm may be different at a point in time from its initial structure when released by the manufacturer, thus making a lawsuit against the manufacturer challenging. Furthermore, liability regarding the use of AI in health care is complicated by the learned intermediary doctrine, which prevents patients from suing medical device manufacturers directly. Under the learned intermediary doctrine, the prescriber, rather than the patient, is considered as the end user of the device. Some legal solutions have been proposed, including conferring personhood to AI devices such that they, not the clinician or the manufacturer, can be sued in case of malpractice lawsuit. The user of the AI device can purchase a liability insurance for the device so that litigations and claims are paid for by the insurance [31-33].

An additional topic raised in the discussion forum is the involvement of for-profit companies in health IT (topic 6; 8% of the corpus), including the impact the algorithms developed by these companies may have on health equity and access to care. Specific AI applications, because of their high cost, may be available or offered only to high-income individuals, thus creating a disparity in access. Also, and not specific to companies, given that algorithms are built with real-world data, their recommendations may replicate the existing biases in patient care and perpetuate prevailing health care inequities [34]. The absence of peer review process for many of the products marketed by these companies raises the question about scientific evidence of the effectiveness and quality of these products [35]. These issues call for oversight by regulatory agencies for AI products developed by these companies.

### AI as a Tool for Disease Screening, Diagnosis, and Treatment

Discussions on the impact of AI disease screening, diagnosis, and treatment elicited many topics with negative average sentiment scores (topic 8: disease screening, topic 9: AI for diabetic retinopathy, and topic 10: AI and medical diagnostic procedure), thus reflecting concerns about the use of AI tools for such purposes. The use of AI for the interpretation of ECG recordings also raised concerns among some commenters. These concerns pertain mostly to the reliability and accuracy of the information output by AI-powered applications. These doubts regarding the accuracy of AI can minimize trust in AI applications and hinder their adoption and use [36].

The issue of privacy of data used to develop these screening and diagnostic tools was largely discussed (topic 5; 8.5% of the corpus). These privacy issues have been raised in previous literature [37-39]. It is often possible to accurately infer personal information from deidentified data [40] or medical images [41]. Hence, legal and ethical regulations governing medical research need to be updated to account for the specific case of big data analytics and medical AI. Furthermore, new techniques have been developed or suggested to protect privacy while developing AI tools. For instance, differential privacy machine learning allows preserving privacy by including random noise into the data such that patterns in the data are preserved, but personal information about individuals is altered [42-44] Federated learning, another privacy-preserving technique, allows developing and testing algorithms across multiple servers using different data sets. Data sets from a given setting are used by a local server and are not shared with other entities; however, the algorithm being developed takes advantage of all the data sets in the network of servers [45-47]. Another approach for minimizing data privacy issues is the use of generative adversarial networks to generate data sets that replicate the statistical distributions of the original data sets and using these generated data sets as training sets for developing the algorithms [48,49].

The advent of AI in health care has been heralded by some commenters as an opportunity for improving care quality by enhancing personalized medicine (topic 11) and clinical tools (topic 3). Specifically, these health care workers highlighted the improved accuracy of image-based diagnostic procedures in radiology, pathology, and other specialties and the potential contributions to precision medicine. This finding corroborates the report by Sarwar et al [50], who found an overwhelmingly positive attitude among a sample of pathologists about the contributions that AI can bring to their specialty, with most of the respondents believing that the technology will increase their efficiency at work. Furthermore, AI can help enhance personalized medicine by better accounting for genetic, physiologic, or lifestyle differences among individuals [44]. Treatment, screening, and diagnostic procedures can then be tailored to the patient, consequently improving their effectiveness.

### Implications

This study has identified some concerns about the adoption of AI in the health care industry that are already being addressed through technological solutions, including patient privacy and security of the data used for developing the algorithms. Other concerns such as the legal ramifications of the use of AI in care operations, existence of biases in the developed algorithms, and quality of those algorithms have already sparked some debates and would be better addressed by involving government officials and public agencies, including agencies responsible for the safety of health care products [51,52]. Challenges that are less discussed currently include the role ambiguity created by the adoption of AI applications and the concerns about health care workers’ job security. AI interferes with the classical mode of care operations in which most of the decision-making and analysis of patient information are performed by health care workers. Ambiguity about the role and tasks of these workers arises when some of the usual decision-making are *delegated* to or complemented by algorithms. Hence, the promotion of AI acceptance in health care should include clarifications about the integration of AI into the traditional diagnostic methods and decision-making processes (history, physical examination, and paraclinical tests) and about health care workers’ job security. It is incumbent upon health care organizations to clearly define the role of AI in their care operations and elicit inputs and comments from their workers to adapt AI adoption plans to the organization. Organizational support to workers is needed for successful adoption of these technologies. Perceived organizational support (measured with items such as “the organization cares about the voice of employees,” “the organization cares about each individual’s well-being,” etc) has been found to reduce the strength of the association between employees’ AI awareness and their turnover intention [19]. Similar findings regarding the positive effect of organizational support on employees’ attitude toward technology have been reported elsewhere [53]. That is, perceived organizational support can mitigate employees’ concerns regarding AI.

### Study Limitations and Recommendations for Future Research

This study has some limitations. First, the health care professionals who made the comments may not be representative of the population of health care professionals in the United States or other countries. In addition, the number of comments collected was relatively low and may not have been sufficiently large to identify a wide range of opinions about AI in health care. Furthermore, the data were collected from a web-based forum that was moderated for civility. Some comments may have been removed by the forum administrator for not respecting the commenting guidelines, thus potentially discarding other viewpoints about the topic of discussion. Finally, the number of topics extracted from the corpus was determined using semantic coherence, held-out likelihood, and exclusivity values. These values suggest only a range of values for the appropriate number of topics, and other methods may yield different values for the number of topics to be extracted. Nevertheless, despite these limitations, this report provides a glimpse into health care workers’ opinions about the use of AI in their job tasks and daily operations of their organizations.

Further studies are needed to investigate each of the topics identified in these discussions. For the topics associated with negative sentiments, studies can be conducted to identify factors or actions that can help dispel or resolve these concerns (eg, training and involvement of health workforce in the selection of AI types).

### Conclusions

AI is predicted to bring dramatical changes in health care organizations’ administrative and patient care routines. The successful implementation of AI-powered technologies requires the involvement of all stakeholders, including patients, health care organization workers, private and public health insurance institutions, and government regulatory agencies. Ethical guidance regarding the role of AI in patient-clinician relationship and legal dispositions clarifying the liability of the health care worker using AI are needed to improve the adoption of the technology by health care organizations.

## Appendix

Multimedia Appendix 1 Quotes from identified topics about the applications of artificial intelligence in the health care industry.

## Abbreviations

AI: artificial intelligence
CMUA: coping model of user adaptation
ECG: electrocardiogram
FREX: frequency and exclusivity
