# Supplementary material for: Artificial Intelligence in Medicine: Text Mining of Health Care Workers’ Opinions
Source: J Med Internet Res. 2023 Jan 27;25:e41138. doi: 10.2196/41138 (PMC9919460; doi:10.2196/41138)
Supplement: Multimedia Appendix 1 [file jmir_v25i1e41138_app1.docx]

**Appendix**. Quotes from identified topics on the applications of artificial intelligence in the healthcare industry

| **Topic/ Label** | **Percentage of corpus** | **Quotes** |
| --- | --- | --- |
| Topic 1: AI replacing humans | 11.6 | 1)” By the time, AI has significant impact on the radiology workforce, a similar impact will also occur on the entire medical workforce. However, no amount of computing will replace the human understanding. Two human beings can make entirely different decisions for the same problem or the same human being can make two different decisions for the same problem at different times and therein lies the beauty of human mind. I don't think the computers will learn to feel the empathy.”  2) “About 30 years ago an attorney friend told me my job would be replaced by computers. We radiologists know computers have a tremendous role and new roles will be coming---but they do not replace judgement which is only a decision the human brain can make.”  3) “Regarding the arguments, whether computers can replace doctors, I think that both sides are correct, but with different qualifications. Suppose that everything a doctor will be able to do, a computer robot will eventually be able to do. If this happens before we have solved all the problems which cause our body's to age, deteriorate and acquire infections, it will require doctors to perform some of the decisions and tasks and relate to the patients. However if our experience and learning process evolves our medical technology gradually, to the state where the body will be able to repair itself without any intervention from doctors, then doctors may no longer be necessary.”  4) “Overall I think this is scary but I am pretty sure there is no computer or software that will ever replace my common sense, instinct, empathy, my eyes, ears and hands, my emotions, and most of all the arts of healing!” |
| Topic 2: AI and classical medical examination | 10.3 | 1) “I say a good history, a physical exam is a must. Adding lab etc. helps to make a diagnosis. Real intelligence just not AI”  2) “I am very much in favor of the standard history and physical exam. A colleague of mine often brags that he no longer performs physical exams because technology has made them a "dinosaur." A close friend of mine almost died because the ER physician did no physical exam and had the scribe adhere closely to a gastroenteritis history protocol. My friend was not allowed to add any information that conflicted with the diagnosis of gastroenteritis. The diagnosis was missed, the appendix ruptured and he became septic. Fortunately, he had an excellent surgeon and hospitalist and he is fine today.”  3) “There is no shortcut to detailed good history and thorough physical exam in the patient - doctor encounters, good differential diagnosis, appropriate investigations and interpretations of the results generated, before making any rational diagnosis with proper therapeutics. Most of the medical teachings are directed at emphasis on using assisted technologies in teaching students and the students are much younger at the time in training and depend on computers, rather than they devoting the time to read in-depth to the subject matter.”  4) “Technology has evolved to augment clinical practice not to replace it .It's evolution has been directed by clinical need . Point of care tests are ever advancing augmenting and expediting the clinical evaluation to enhance outcomes but targeted application of these tests is not possible with the all-important foundation steps of the process: history and examination. Let's not throw the baby out with the bath water - the process/ritual has worked for years . Students need to be taught the importance of physical examination in patient assessment , to treat the patient and not the test .” |
| Topic 3: AI as a clinical tool | 10.1 | 1) “We need to either give intelligent, experienced clinicians all the clinically relevant information with minimal time and effort on their part AND the time to make the diagnosis. This is a crisis in our medical care especially for the sickest patients. Otherwise better algorithms as showcased on Sixty minutes with the IBM Watson computer for oncology therapy will be needed to assist our beleaguered primary care and specialist colleagues who do not have the time or incentive to do the job optimally.”  2) “I happen to be an enthusiast for IBM's Watson and other cyberassistance to the physician and nurse for the benefit of patients. But, in simple arithmetic, replacing a good human professional who has human limitations with a good AI device that has machine limitations is just a change in the nature of the limitations without true advance. If we integrate their strengths to decrease the total limitations, THAT will be advance.”  3) “Granted the AI has won the spot the cancer photo test- but that does not mean it can outdo the human brain which made it! I am sure the expert dermatologist given the real life test would have performed similar if not better than the machine since a dermatoscope is just a tool that he uses for diagnosis.”  4) “We are always going to see something different and challenging , that is the nature of life and medicine. That is the reason for specialties and integrated care. Augmented Intelligence is again just a TOOL and will not be a solution. While there is no solution to the paradox, there is a methodology to the challenge…grounded in clinical experience and problem solving . I believe this methodology is the outcome of a medical education and clinical experience that developed critical thinking skills that then develop the skills for a meaningful Examination and clinical practice.” |
| Topic 4: Patient care by healthcare professionals | 9.5 | 1) “As a nurse in various patient care settings for more than forty years, I can testify there is both diagnostic as well as therapeutic value to a doctor who listens carefully to a patient. In a fifteen minute time allotment in which a young physician's eyes and brain are more focused on his or her computer screen, key information, both verbal and non, can be missed.”  2) “My take-away from this program was as both clinician (NP-now retired) and patient. I see AI both helping and hindering the process of patient care. Obtaining labs, imaging, and procedural information from the computer is helpful to the clinician that reads and interprets the information prior to seeing the patient. For that same care provider to then have to spend hours inputting that data into progress notes is truly making healthcare actually computer care.”  3) “I am ready for it. It would be very beneficial for the bedside nurse. When we report we use a specific form of reporting. One step is to provide an idea, or our thoughts on what we think would help the patient situation. We do not diagnose, but must be involved in direct patient care. A quick review of symptoms, the client diagnosis , and groupings of related topics. I would try it.”  4) “As a patient, I find it quite frustrating that the doctors do not take the time to listen. Even the PA and NP are being rushed to see more patients. All care providers are thus spending less time with each patient and not hearing what is being said. Since my care is all in a single healthcare system that utilizes a single computer system, I am particularly frustrated that I am required to fill out the same questionnaires for each visit with a physician/specialist.” |
| Topic 5: Data Privacy | 8.5 | 1) “Deidentifying data could be questionable. People with certain rare conditions would be rapidly identified. The same computers that correlate the data could reverse the process. Perhaps it would not identify you specifically but it would categorize you and you would reap the benefits which would include unwanted ads and solicitations. The department of defense considers medical information Personal Identifiable Information (PII).”  2) “I am happy to hear about this disruptive and likely helpful effort. I do have concerns about whether the use of this data in this way opens the door to creation and storage of protected health information (PHI). Simply saying people who value privacy “wouldn’t be on social media” is not adequate. If the type and amount of data is sufficient to meet the standard, proper observation of HIPAA compliance is required according to the Privacy Rule (see below). This includes informing the users of that data is being kept, allowing access (a particular concern with current methodology), and following appropriate procedures prior to disclosing information about their mental health.”  3) “The company I don’t trust with my internet searches now has access to my formerly protected health information. How long before they start selling this info to insurance companies in the name of capitalism. Then insurers will cancel my policy claiming they reached their decision using proprietary data models they won’t share.”  4) “Way too Big Brother for me. Who collects the data? Who has access? Who decides which fitness trackers to follow? Are the wearers notified BEFORE data collection begins? If you opt out, is data collected anyway? Can you opt out? How is collected data used, beyond notifying someone that you MAY have COVID? This is a slippery slope. Today we collect heart rates for COVID monitoring, then your insurance company denies your claim because you are not active enough. Or your car insurance denies your claim because you didn't sleep enough. No thanks!” |
| Topic 6: For-profit companies involvement in health information technology | 8.0 | 1) “In addition to potential pitfalls it's important to see the benefits of the human level AI (will get there probably in "millennial physician's" lifetime) in healthcare and how it can transform the access and affordability of healthcare . That is if the government and university leaders do not get involved today in creating a health AI system that would benefit all, this will become an exclusive domain of Google and Amazon (already working on it) which will mean more profit for them, and less healthcare equality. In my mind AI is the solution to healthcare cost and access disparity but the problem will get worse if tech giants will have sole control of it.”  2) “When mentioning surveillance and who inspects the machine, don't forget about the Big Tech censors that hang out in the public space like Google, Facebook, Twitter, Government and others that can have inhibiting affects for change to be sure. Public understanding of what is and isn't being in the healthcare arena is not too great. Those within the system know the everyday truth.”  3) “It should also be noted that the VA software is in the public domain and is available for outside development, although few private enterprises have taken advantage of this. By its nature, an open-source system promises lower profits to those touting it than a copyrighted commercial system. And yet—-what is an EHR for, anyway? To code rapaciously to garner those RVU’s, or to serve as a facile communication tool between clinicians and other members of the healthcare team. I vote enthusiastically for the latter. If CPRS/VistA is flawed, there is genius in the IT community to fix it and continue incorporating those features that made it the favorite of Physicians into a widely-disseminated EHR. Let’s not devolve to #10 or #20 because someone wants to wring all the profit they can out of it, at the expense of Physician burnout.”  4) “Interesting , a prediction by a Venture Capitalist! In my lifetime I have seen medicine change from a respected , caring profession to a profit oriented , self-marketing service industry . I'm saddened and embarrassed every time I see a billboard for some hungry medical entity .” |
| Topic 7: AI for cardiac monitoring | 7.7 | 1) “The benefits of the Apple Watch go far beyond its new found ability to check for arrhythmia. They also enable older patients to call for help anytime and anywhere at the push of a button and, in fact, will make the call automatically if the person falls and does not get back up within a minute -- even in the shower.  But, even more importantly: It encourages and monitors daily physical activity while discouraging prolonged sitting. In the overall scheme of things, checking for arrhythmia, while an admirable feat of technology, is a minor feature of how this device promotes and protects health.”  2) “While I suspect that you are correct that "smart watches" have been mostly sold to younger populations, the Apple Watch is increasingly appropriate for seniors. That is:  As you point out, its ability to continuously monitor for rhythm disturbances benefits the older population.  But also: It can also act as a water proof cell phone that can be worn continuously and is capable of calling for help at the push of a button.  And also: It has fall detection and, if the person falls but does not get up within a minute, the Apple Watch automatically dials 911 and simultaneously the texts the emergency contact.  Essentially, it has (or soon will) replaced all those "I've fallen and I can't get up" devices that older people hang around their necks -- and remove when they take a shower.”  3) “I am amused that as we have sped to market wearable devices that will help with a cryptic diagnosis of asx AND paroxysmal AFib (which is indeed serious, under-diagnosed, and fairly common), we, Apple and Fitbit are still stumbling along in a fog. Need the diagnosis ? An implanted loop recorder will not be bested for a long time. The Apple Watch is a screening device and like most screening tools in low prevalence groups, they add more problems than they solve.”  4) “As someone that lives with AFib I recently was able to ascertain its accuracy and it was accurate to the beat versus the ER/hospital monitor. Generally I know when I am in Afib but the watch definitely did validate it and it also tracked my HR swings. Personally there should be more that can be done with these types of devices that HC professionals should get up to speed about and possibly give them more information to personalize medicine. In my opinion every Afib patient should have an Apple Watch.” |
| Topic 8: Disease screening | 7.1 | 1) “I'm sure this system will require calibration, but I fear that its main accomplishment may be to magnify the already excessive number of false positives which, in the wrong hands (and those are many), will lead to lot more unnecessary perforations, mangled colons, and deaths. Of course preventing cancers is the ultimate goal, but there is a balance in this business, and I'm not sure this is going to make it better for overall morbidity and mortality.”  2) “this test is not highly reliable. They kept narrowing down their sample size, excluding the more educated and such. Hence, their result only fits a narrow subset. And it is, at best, 70% accurate. So that raises the issue of false positive. When a person who will not get Alzheimer but is told they will get Alzheimer, what effect does that have?”  3) “It is not humans vs machines , it is more about humans + machines vs problems. Indeed we have a lot of work to improve the high false positive rates that lung cancer screening programs have reported. Most of patients are diagnosed as stage IV lung cancer and although huge changes have arrived in the last decade with the introduction of target therapies and immunotherapy, we should increase the rate of early lung cancer which has a curative intent. Which other companies do you know that offer this product based on AI ?”  4) “This is a cool study! But it does not sound like a terribly useful test. The base rate of conversion to cognitive impairment and Alzheimer in the sample was 50% (40-40), so random guessing hold yield 50% accuracy. The linguistic analysis boosted that to 70%, representing a statistically significant effect but not one you’d rely on in clinical work. And since the base rate of incipient dementia in a typical clinic is way below 50% here would be lots and lots of false positives for every case found.” |
| Topic 9: AI for diabetes retinopathy | 7.0 | 1) “Was a cost analysis done for comparison with the current standard practice of a minimum of yearly visit of every diabetic to the ophthalmologist? This newly FDA approved screening method is mislabeled as artificial intelligence; there is no algorithm to the process, it is simply the same digital picture exam that the ophthalmologist performs and does not qualify as artificial intelligence.”  2) “So we add extra cost to the system as this so called screen by AI identifies only 87% so what happens to 10-13% missed. In my practical experience I had patients who were pre diabetic develop eye problems (retinal DR lesions)strange but documented. All diabetics must be refereed to ophthalmologist for careful retinal evaluation. 900 patient data is not large and this may give rise to false sense of security and lead to more blindness.”  3) “The algorithm also may hinder appropriate education and targeted intervention for patients with milder diabetic retinopathy. The LEADING cause of vision loss in diabetes is diabetic macular edema (DME), the gold-standard for detection of which is spectral domain optical coherence tomography (sdOCT), NOT a non-mydriatic retinal photo - most eye care providers, optometrists and ophthalmologists alike, have and routinely use sdOCT.”  4) “The proposed camera IDx-DR device will have benefit to discover diabetic retinopathy (BDR) in the above described population. If the healthcare program will accept to use such a device as an ambulatory system, to arrive in areas with no eye doctors and make examinations in primary care clinics this will be the benefit. Any person detected with BDR will need a further mandatory eye specialist examination with pupil dilatation. The benefit will be: the person himself, the diabetes specialist , to decide further treatment strategy and the medical care assistance to save more expenses of care with a more handicapped of sight member.” |
| Topic 10: AI and Medical diagnostic procedure | 6.8 | 1) “Much of the statements in the article look like wishful thinking. 'Trust' and 'relating' to patient doesn't help make a diagnosis. Though these are important, we all know the salary clinicians get is for making a diagnosis and shelling out prognosis, investigation and treatment. That being said, diagnostics and prescription treatment can be easily overtaken by AI.”  2) “So we are going to make medical care more human by taking doctors out of the diagnosis and treatment decisions? And only one diagnosis and treatment prescribed by a machine with no chance of getting a second opinion? Sure just trust the experts that programmed the machine. What could possibly go wrong??? And who wants a bunch of sensors implanted in their body? RFID chips anyone???”  3) “I think having a dermatologist check EVERY AI diagnosis is crucial. The diagnosis can be affected by photo quality, code corruption, and a host of other issues. Visual screening is tailor-made for AI systems. More difficult will be the subtle clues physicians rely upon daily in making diagnoses -- breath smell, body odors, skin color, elasticity, dehydration, etc. And, of course, the myriad behavioral "tells" that give away both some somatic and psychosocial problems.”  4) “Lung sounds are diagnostically helpful when they are typical (a rather uncommon occurrence). More often than not they do not equate with a specific diagnosis but rather point us to some direction, and we need further information (usually from imaging and other techniques) to further define our auscultatory findings. New technologies may add something to this diagnostic process but at the end of the day we should rely on our composite evaluation of the patient by history and examination in the light of past experience. If we cannot do that, then computer-analyzed lung sounds may simply add another factor of frustration in our practice.” |
| Topic 11: Personalized medicine | 6.8 | 1) “Until technology can collect a family history, and assess full nutritional intake and supplement use, lifestyle, symptomology, etc., patient reported data will remain critical to the practice of medicine. Eventually, combining a full range of omics data with patient reported data will herald true personalized and precision medicine.”  2) “As a physician interacting with patients, we make those perceptions and decisions on the fly and adapt our interviews to suit the patient's educational, ethnic, and intellectual capacity. Machine learning that is brighter than any individual practice should be able to improve differential diagnosis lists and should be able to assist with work up that makes sense. When that is possible, the cost of healthcare should be more controllable.”  3)” Technology is forever evolving, scientist are forever discovering, the role of the GP forever changing. Unfortunately the patient is forever changing, it is called ageing in a forever changing environment. Scientist identify diseases etc., previously not able to be identified but have lurked in the human. This is with the aid of advancement of technology. Add medications, treatments and surgeries forever changing. All is the future of healthcare.”  4) “When I told my doctor I ran for an hour at 90% max heart rate -- he has no idea what that means or how to deal with it. He needs to find out. When I provided my nutritionist with a report of what I ate for a month, she had no idea how to deal with it. She needs to find out. This technology will, more than any other initiative, will enable the patient to manage their own health and to turn the healthcare industry to becoming patient centric. The truth is: Healthcare will become patient centric -- the question is: will the healthcare professional be a part of it?” |
| Topic 12: AI for patient ECG interpretation | 6.7 | 1) “I diagnosed my own AFib with the Alivecor device prior to the watch having that capability. It was confirmed by a cardiologist and I was put on apixaban. No Holter nor office ECG has ever caught more than PACs in me. Without the Alivecor, I probably would have had to have the implanted monitor to confirm what was happening.”  2) “I am not a cardiologist, and had been working in close in ER in Sudan for 9 years, Oman 4 years and Saudi Arabia since 1997. I was lucky may be and diagnosed AFib using only stethoscope and ECG come thereafter. Listening to HS and grossly irregular pattern leads you to it. I used to read the interpretation after my own reading and asking questions to the patient and family.”  3) “I over-read ECG as a cardiologist and definitely found that one of the best "learning" experience was reading the manufacturers document on the ECG analysis program which gives a thorough explanation of how the computer analysis is done. A few hours well invested. The "overreading" of ECG has never been the same: one becomes a better over-reader! All of a sudden you understand the reason why incorrect readings occurs.”  4) “I have been reading ECG for 40 years and the computer is good, but not equipped to handle subtle findings like Epsilon waves, hyperkalemia, AFib in the setting of artifact, early MI or artifact in general. It may help a generalist, but it needs to be over-read to prevent serious errors. Anyone with computer can send their ECG to a friendly cardiologist to avoid problems.” |
